# Moderate-intensity treadmill running relieves motion-induced post-traumatic osteoarthritis mice by up-regulating the expression of lncRNA H19

**DOI:** 10.1186/s12938-021-00949-6

**Published:** 2021-11-18

**Authors:** Xuchang Zhou, Hong Cao, Miao Wang, Jun Zou, Wei Wu

**Affiliations:** School of Kinesiology, Shanghai University of Sport, Shanghai, China

**Keywords:** Post-traumatic osteoarthritis, lncRNA H19, Subchondral bone, Cartilage, Treadmill running

## Abstract

**Background:**

The purpose of this study was to explore whether moderate-intensity exercise can alleviate motion-induced post-traumatic osteoarthritis (PTOA) and the expression change of lncRNA H19 during this progression.

**Methods:**

Twenty-week-old male C57BL/6 mice were randomly divided into five groups: model control group (MC group, *n* = 6), treadmill model group (M group, *n* = 6), rehabilitation control group (RC group, *n* = 6), treadmill model + rehabilitation training group (M + R group, *n* = 6) and treadmill model + convalescent group (M + C group, *n* = 6). Paraffin sections were used to observe the pathological changes in the mouse knee joint in each group. A micro-CT was used to scan the knee joint to obtain the morphological indexes of the tibial plateau bone. Real-time PCR was used to detect the mRNA levels of inflammatory factors, synthetic and catabolic factors in cartilage.

**Results:**

After high-intensity exercise for 4 weeks, the inflammation and catabolism of the mouse knee cartilage were enhanced, and the anabolism was weakened. Further study showed that these results were partially reversed after 4-week moderate-intensity training. The results of hematoxylin–eosin staining confirmed this finding. Meanwhile, high-intensity exercise reduced the expression of lncRNA H19 in cartilage, while the expression of lncRNA H19 increased after 4 weeks of moderate-intensity exercise.

**Conclusion:**

High-intensity treadmill running can cause injury to the knee cartilage in C57BL/6 mice which leads to PTOA and a decrease of lncRNA H19 expression in cartilage. Moderate-intensity exercise can relieve PTOA and partially reverse lncRNA H19 expression.

## Background

Studies have shown that the knee cartilage bears about three times the body’s weight while performing daily activities, about 10 times the body’s weight during running, and about 20 times the body’s weight when participating in jumping [1–4]. Under normal circumstances, chondrocytes are subjected to mechanical loads of millions of cycles per year [5]. However, an abnormal mechanical load that can be caused by factors such as obesity, joint instability, and excessive load increase in the activities of daily life may cause cumulative micro-injuries or trauma to the joints, and induce the conversion of healthy cartilage to degenerative joint disease phenotype-osteoarthritis (OA) [5–7]. Post-traumatic osteoarthritis (PTOA) is an important subtype of OA, which is more common in adolescents, and is usually closely related to acute sports injuries or excessive exercise load [8]. It is believed that mechanical stimulation can affect the biosynthesis of chondrocytes and alter cartilage morphology [9]. Previous studies have shown that there is a biomechanical "window" that is required to maintain cartilage homeostasis. A “too high” or “too low” load can cause a cartilage lesion [10], while the appropriate load intensity can inhibit osteoclast activity in the subchondral bone, slow down the degeneration of the articular cartilage and prevent the development of PTOA. Numerous animal experiments have verified that treadmill running can relieve and treat PTOA. This relief includes a decline in Mankin scores and OARSI scores of the articular cartilage, reduced bone mass loss of the subchondral bone, inhibition of proinflammatory factor production such as interleukin-6 (IL-6) and tumor necrosis factor-α(TNF-α), and increased IL-10 secretion to reduce joint inflammation. Also, moderate-intensity exercise can inhibit the expression of matrix metalloproteinases (MMPs), and therefore reduce the death of articular chondrocytes and matrix degradation [11]. Therefore, in animal experiments, moderate-intensity treadmill running is often used in research on the therapeutic mechanism of PTOA, [12, 13] while high-intensity treadmill running is often used in research on the model of PTOA [14, 15].

Although more than 80% of the human genome is transcribed, less than 2% of the human genome is transcribed as mRNA, and most of the rest is transcribed as non-coding RNAs (ncRNAs). Long non-coding RNAs (lncRNAs) are ncRNAs that have a length of over 200 nt and have no protein-coding function [16]. LncRNAs are widely distributed in the human genome [17]. Previous studies have focused on protein-encoded RNA, rather than on non-coding RNA, which is thought to be the “junk RNA” or “transcriptional noise”. Unlike microRNAs (miRNAs), lncRNAs can be folded into complex secondary or higher spatial structures to better identify targets [18]. With the development of high-throughput sequencing technology in recent years, more studies have shown that lncRNAs can regulate the expression of protein-coding genes in epigenetics, transcription, and post-transcription, and therefore affect a series of biological processes [19–21].

LncRNA H19, located in human chromosome 11p15.5, is one of the most famous imprinted genes and is only transcribed from a maternally inherited allele [22]. LncRNA H19 is highly expressed during the period of embryonic development, however, after birth, its expression is decreased in tissues other than skeletal muscle. It only increases again in a few tumor tissues [23–25]. LncRNA H19 is highly conserved in the evolutionary process and has a very low mutation rate in exons, which indicates that it has important biological functions [26]. Studies have found that the high expression of lncRNA H19 is closely related to the proliferation, differentiation, and metastasis of tumor cells, [27] and the expression of lncRNA H19 is significantly correlated with the severity of tumor invasion [28]. In addition, lncRNA H19 is also involved in the regulation of atherosclerosis, [29] myocardial injury, [30] skeletal muscle regeneration, [31] and osteogenic differentiation [32]. Recent studies have shown that 73 lncRNAs were up-regulated and 48 lncRNAs were down-regulated in OA cartilage when it was compared with normal cartilage by microarray analysis. Among them, 21 lncRNAs, including lncRNA H19, were up-regulated by more than two times the normal level [33]. The subsequent RT-PCR further confirmed that pathological changes in OA may be related to the abnormal expression of lncRNAs. At present, several studies have confirmed that lncRNAs play an important role in OA lesions [34]. Steck et al. [35] studied the relationship between lncRNA H19 and the development of OA lesions. In this study, the expression of lncRNA H19 decreased in a culture environment with enhanced chondrocyte catabolism. Conversely, the expression of lncRNA H19 increased in a culture environment with enhanced chondrocyte anabolism. LncRNA H19 may be closely related to chondrocyte metabolism. In a normal culture environment without the induction of inflammatory factors, the levels of expression of lncRNA H19 and COL2A1 were significantly correlated, while in a culture environment induced by inflammatory factors, the levels of expression of lncRNA H19 and COL2A1 were not significantly correlated, which indicates that lncRNA H19 and COL2A1 were not simply co-regulated in the inflammatory environment [35]. However, current studies on the relationship between lncRNA H19 and OA are only focused on the cellular level, and further studies need to identify the specific regulatory mechanism of lncRNA H19 on OA. This study is based on the clinical experience of exercise therapy in the treatment of PTOA and the early stage experimental knowledge of our team. We induced a mouse PTOA model through high-intensity treadmill running, and then performed rehabilitation training on the PTOA mice with moderate-intensity treadmill running. We noted the changes in the expression of lncRNA H19 during the modeling and rehabilitation training in order to provide a reference for the related research on sports treatment of PTOA.

## Results

### Weight changes

**Fig. 1:**
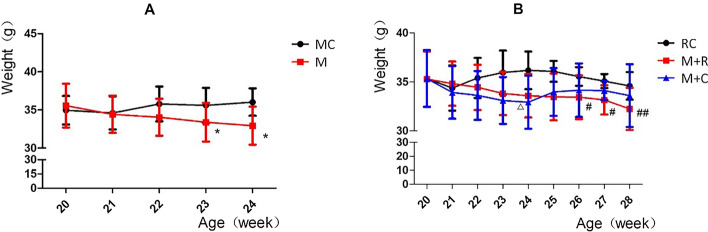
**A** Weight changes in MC group and M group, **B** Weight changes in RC group, M + R group, and M + C group. (MC group: model control group, M group: treadmill model group, RC group: rehabilitation control group, M + R group: treadmill model + rehabilitation training group, M + C group: treadmill model + convalescent group. *N* = 6, *P < 0.05, intra-group comparison of M group;^△^*P* < 0.05, intra-group comparison in M + C group;^#^*P* < 0.05, ^##^*P* < 0.001.)

As exhibited in Fig. 1A, the weights of the mice in the MC group fluctuated dynamically as the mice aged but without significant change. During the modeling period of high-intensity treadmill running, the weights of the mice in the M group (treadmill model group) continually declined, and there was a statistical difference when the mice were 23 weeks old (fourth week of treadmill running), compared with 20 weeks old (before treadmill running). Also, at the end of the modeling period (mice were 24 weeks at this time), the mice in the M group weighed less than those in the MC group (model control group), but there was no statistical difference. As shown in Fig. 1B, the weight change in the RC group (rehabilitation control group) was almost the same as that in the MC group, and also showed dynamic fluctuation without any significant change. The weights in the M + R group (treadmill model + rehabilitation training) continued to decrease during the period of high-intensity and moderate-intensity treadmill running, and there was a statistical difference at the age of 26 weeks. The weight in the M + C group (treadmill model + convalescent group) showed a continuous trend of weight loss during the high-intensity treadmill running. However, the weight in the M + C group showed a trend of regaining weight in the first week after the end of the modeling period (mice were 25 weeks at this time). At the end of rehabilitation training (mice were 29 weeks at this time), the weights of mice in the M + R group and the M + C group were lower than in the RC group, but there was no statistical difference.

### Pathological changes

#### Pathological changes of the mice knee joints after high-intensity treadmill running

**Fig. 2:**
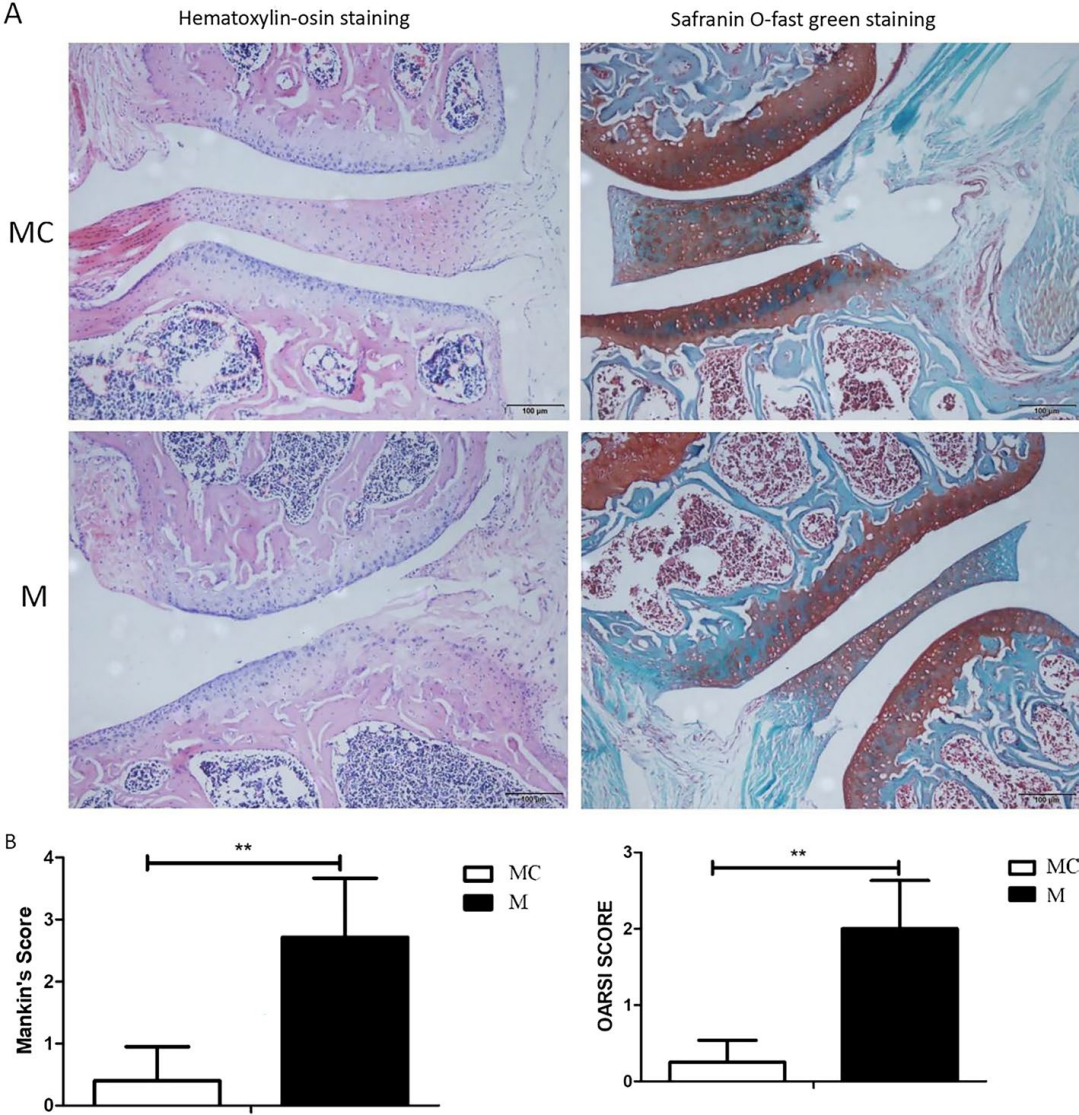
The effect of high-intensity treadmill running on the cartilage of mice knee joints (**A** sections of the knee joint of the high-intensity treadmill running mice with hematoxylin–eosin staining and saffron O staining under the 200-fold light microscope; **B** Mankin and OARSI scores of hematoxylin–eosin staining and saffron O staining in the knee joint of high-intensity treadmill mice; MC group: model control group, M group: treadmill model group, *N* = 6, **P < 0.01, Mankin scores;^##^*P* < 0.01, OARSI score)

The paraffin sections of the knee joints of the mice were stained with hematoxylin–eosin and safranin O–fast green staining, respectively. The stained sections were observed and photographed using a 200 Olympus inverted microscope. The hematoxylin–eosin staining results showed that when compared with the MC group, the articular surface of the knee joint in the M group was rough, with a few irregular cracks on the surface and a decreased number of chondrocytes. Mankin scores indicated that there was a significant statistical difference in the pathological changes of the cartilage. The fast green staining results showed that the knee joint in the MC group had a smooth surface, while the knee joint in the M group had obvious cracks, and the hyaline cartilage layer in the M group was significantly thinner than in the MC group, with significant cartilage loss. There was a significant difference in the OARSI score between the two groups (Fig. 2).

#### Pathological changes of mice knee joints after moderate-intensity rehabilitation training

**Fig. 3:**
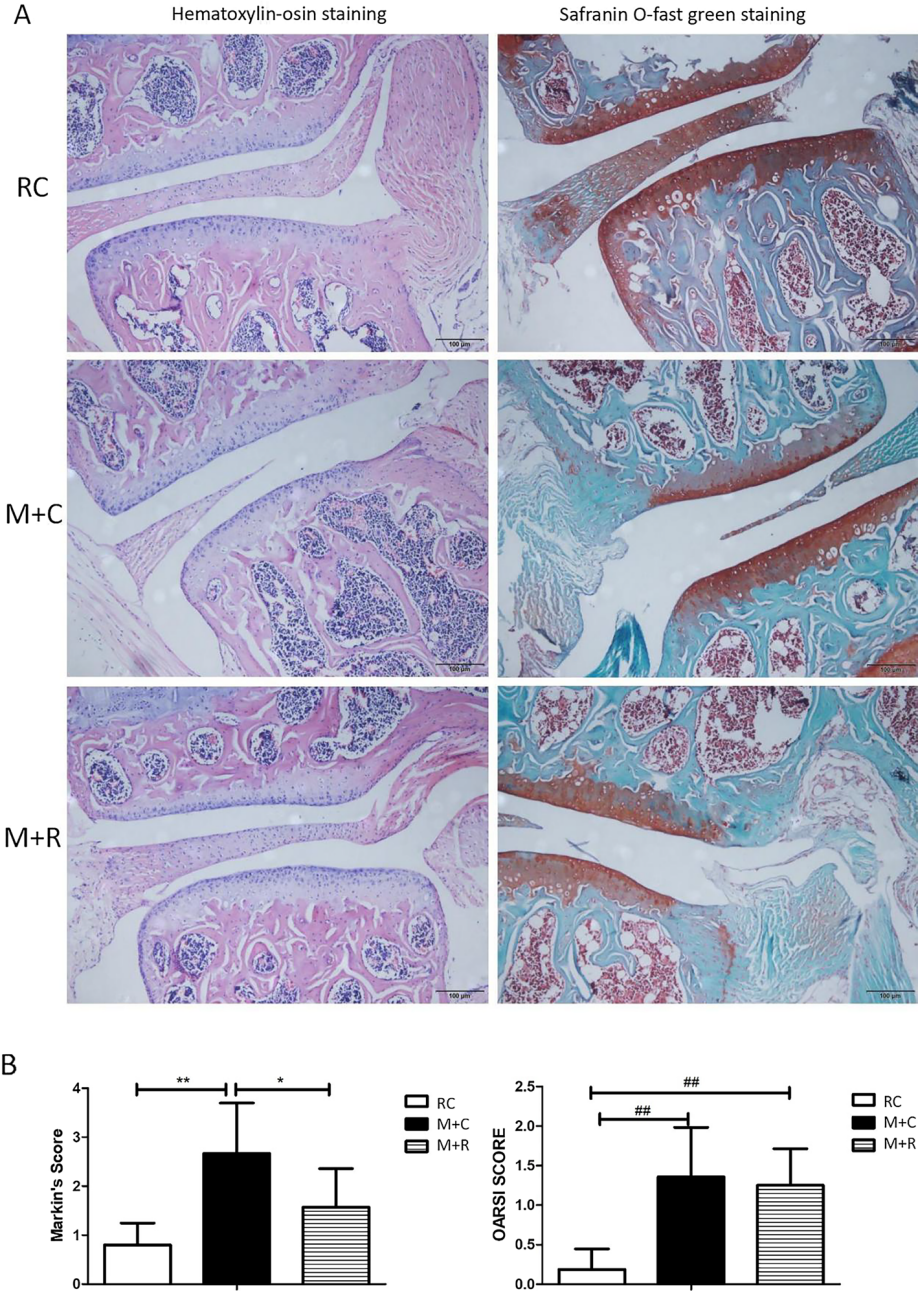
Effects of moderate-intensity treadmill rehabilitation training on mice knee cartilage (**A** section-diagram of moderate-intensity treadmill running mice knee cartilage with hematoxylin–eosin staining and safranin O–fast green stain under the 200-fold light microscope; **B** Mankin and OARSI score of hematoxylin–eosin staining and safranin O–fast green stain of the knee cartilage of mice with moderate-intensity treadmill running. RC group: rehabilitation control group, M + R group: treadmill model + rehabilitation training group, and M + C group: treadmill model + convalescent group. *n* = 6, *P < 0.01, **P < 0.01, Mankin scores; ^##^*P* < 0.01, OARSI score)

The results of the hematoxylin–eosin staining showed that the articular cartilage surface of the RC group was smooth without cracks, the chondrocytes were evenly distributed and neatly arranged, and the tidal line was complete and visible. In the M + C group, there were irregular defects on the articular cartilage surface of the knee joint, the cartilage was somehow lost, and the distribution of chondrocytes was uneven and there was a decreased number of chondrocytes. When compared with the M + C group, the cartilage surface of the knee joint in the M + R group was smoother, the cartilage layer was thicker, and the distribution of the chondrocytes was normal. The results of the Mankin score showed that the pathological changes of the knee cartilage in the M + R group were less severe than in the M + C group, and the difference was significant. The degree of the pathological changes of the knee cartilage in the M + R group was more serious than the changes in the RC group, but there was no significant difference. The safranin O–fast green stain showed that there was no obvious crack in the cartilage in the M + R group. Staining also showed that some cartilage fibrosis and volume loss were found in the M + R group when compared with the RC group, which indicates that the pathological changes in the cartilage in the M + R group were more serious than in the RC group, and the OARSI score of the two groups was significantly different. When compared with the M + C group, the pathological changes of the knee cartilage in the M + R group were less severe, but there was no significant difference in the OARSI score (Fig. 3).

### Morphological changes of the subchondral bone of the knee joints in mice

#### Morphological changes of the subchondral bone of knee joints in mice after high-intensity treadmill running

**Fig. 4:**
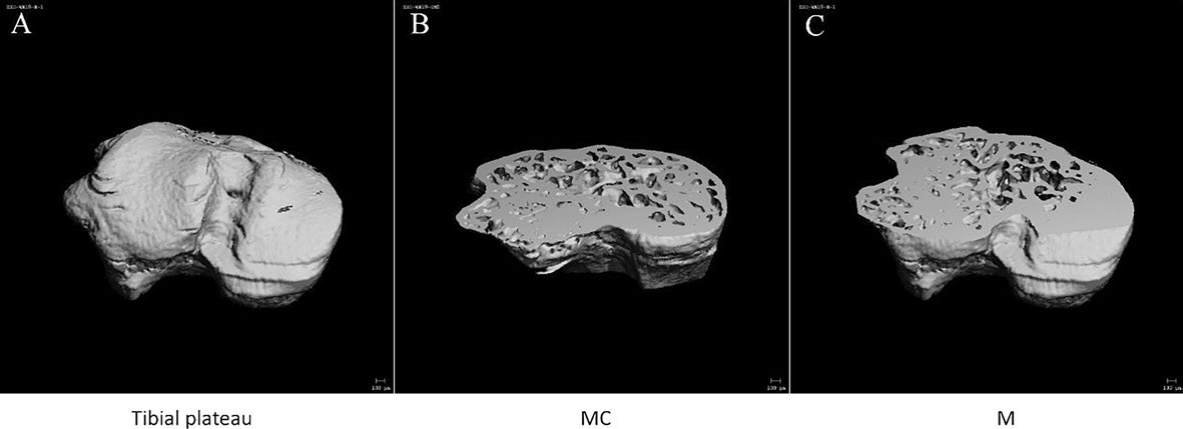
Morphological changes of tibial plateau bone in mice of MC group and M group (**A** three-dimensional (3D) reconstructed images of tibial plateau; **B** the image of tibial plateau of MC group was sectioned along the horizontal plane, from the top at 40%; **C** the image of tibial plateau of M group was sectioned along the horizontal plane, from the top at 40%. MC group: model control group, M group: treadmill model group)

After scanning the knee joint, the medial and lateral subchondral bones of the tibial plateau were reconstructed separately to obtain the morphological indexes of the subchondral bone of the tibial plateau. The results showed that the BMD, BV/TV ratio, and Tb.Th of the LM group was significantly higher than those in the LMC group. The above data in the MM group were also higher than in the MMC group, but there was no significant difference (Fig. 4 and Table 1).

**Table 1:** Morphological changes of the subchondral bone of the medial and lateral tibial plateau in mice after high-intensity treadmill running

Group	BMD(mg HA/ccm)	BV/TV	Tb.N	Tb.Th	Tb.Sp
LMC	922.77 ± 4.282	0.544 ± 0.187	7.297 ± 0.218	0.087 ± 0.003	0.130 ± 0.005
LM	958.425 ± 18.716*	0.583 ± 0.031*	7.032 ± 0.349	0.100 ± 0.005*	0.131 ± 0.007
MMC	940.886 ± 5.236	0.619 ± 0.0226	7.199 ± 0.218	0.109 ± 0.005	0.133 ± 0.004
MM	974.429 ± 22.776	0.668 ± 0.033	7.131 ± 0.253	0.127 ± 0.139	0.130 ± 0.011

Comparison of BMD, BV/TV, Tb.N, Tb.Th and Tb.Sp of the subchondral bone of the medial and lateral tibial plateau in mice. LMC group: MC group lateral tibia plateau, LM group: M group lateral tibia plateau, MMC group: MC group medial tibia plateau, MM group: M group medial tibia plateau, *n* = 3, *P < 0.05, LM group vs LMC group

#### Morphological changes of the subchondral bone of knee joint in mice after moderate-intensity treadmill running

**Fig. 5:**
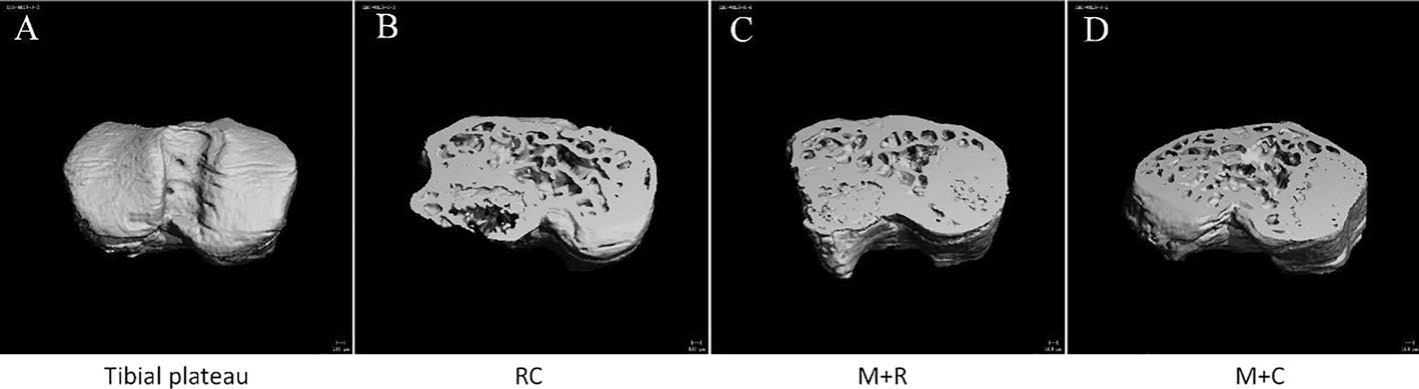
Morphological changes of tibial plateau bone in mice of RC group, M + R group, and M + C group (**A** three-dimensional reconstructed images of tibial plateau; **B** the image of tibial plateau of RC group was sectioned along the horizontal plane, from the top at 40%; **C** the image of tibial plateau of M + R group was sectioned along the horizontal plane, from the top at 40%; **D** the image of tibial plateau of M + C group was sectioned along the horizontal plane, from the top at 40%. RC group: rehabilitation control group, M + R group: treadmill model + rehabilitation training group, M + C group: treadmill model + convalescent group)

In the lateral tibial plateau of subchondral bone, the BMD, BV/TV ratio, Tb.N, and Tb.Th of the M + R group mice were higher than in the RC group when compared with the RC group, and only the difference of the BMD was statistically significant. The BMD and Tb.Th values of the lateral tibial plateau of the mice in the M + C group were significantly higher than those in the RC group; in the medial tibial plateau of subchondral bone, the BMD values of the M + C group were higher than the RC group and showed a statistical difference. Meanwhile, when compared with the RC group, there was no significant difference in the increase of BMD, BV/TV ratio, Tb.N, and Tb.Th and the decrease of Tb.Sp of the M + R group mice, compared with the RC group, there was no significant difference (Fig. 5 and Table 2).

**Table 2:** Morphological changes of the subchondral bone of the medial and lateral tibial plateau in mice after moderate-intensity treadmill running

Group	BMD(mg HA/ccm)	BV/TV	Tb.N	Tb.Th	Tb.Sp
LRC	950.925 ± 27.040	0.5712 ± .033	7.166 ± 0.441	0.094 ± 0.008	0.131 ± 0.004
LM + R	999.071 ± 15.379*	0.5734 ± 0.037	7.370 ± 0.236	0.101 ± 0.010	0.128 ± 0.010
LM + C	987.680 ± 5.319*	0.629 ± .0117	6.951 ± 0.375	0.111 ± 0.005*	0.134 ± 0.011
MRC	969.248 ± 21.332	0.616 ± 0.056	7.094 ± 0.337	0.104 ± 0.017	0.139 ± 0.013
MM + R	1008.845 ± 4.041	0.647 ± 0.054	7.655 ± 0.318	0.119 ± 0.011	0.123 ± 0.011
MM + C	968.176 ± 4.204^##^	0.572 ± 0.016	7.214 ± 0.268	0.092 ± 0.001	0.127 ± .006

Comparison of the BMD, BV / TV, Tb.N, Tb.Th and Tb.Sp of the subchondral bone under the medial and lateral tibial plateau in mice. (LRC group: RC group lateral tibia plateau, LM + R group: M + R group lateral tibia plateau, LM + C group: M + C group lateral tibia plateau, MRC group: RC group medial tibia plateau, MM + C group: M + C group medial tibia plateau, MM + R group: M + R group medial tibia plateau. *n* = 4, *P < 0.05, LM + R group or LM + C group VS LRC group, ^##^*P* < 0.01, MM + C group VS MRC group)

### mRNA expression changes in the knee cartilage in mice

#### Changes in mRNA expression of the knee cartilage in mice after high-intensity treadmill running

**Fig. 6:**
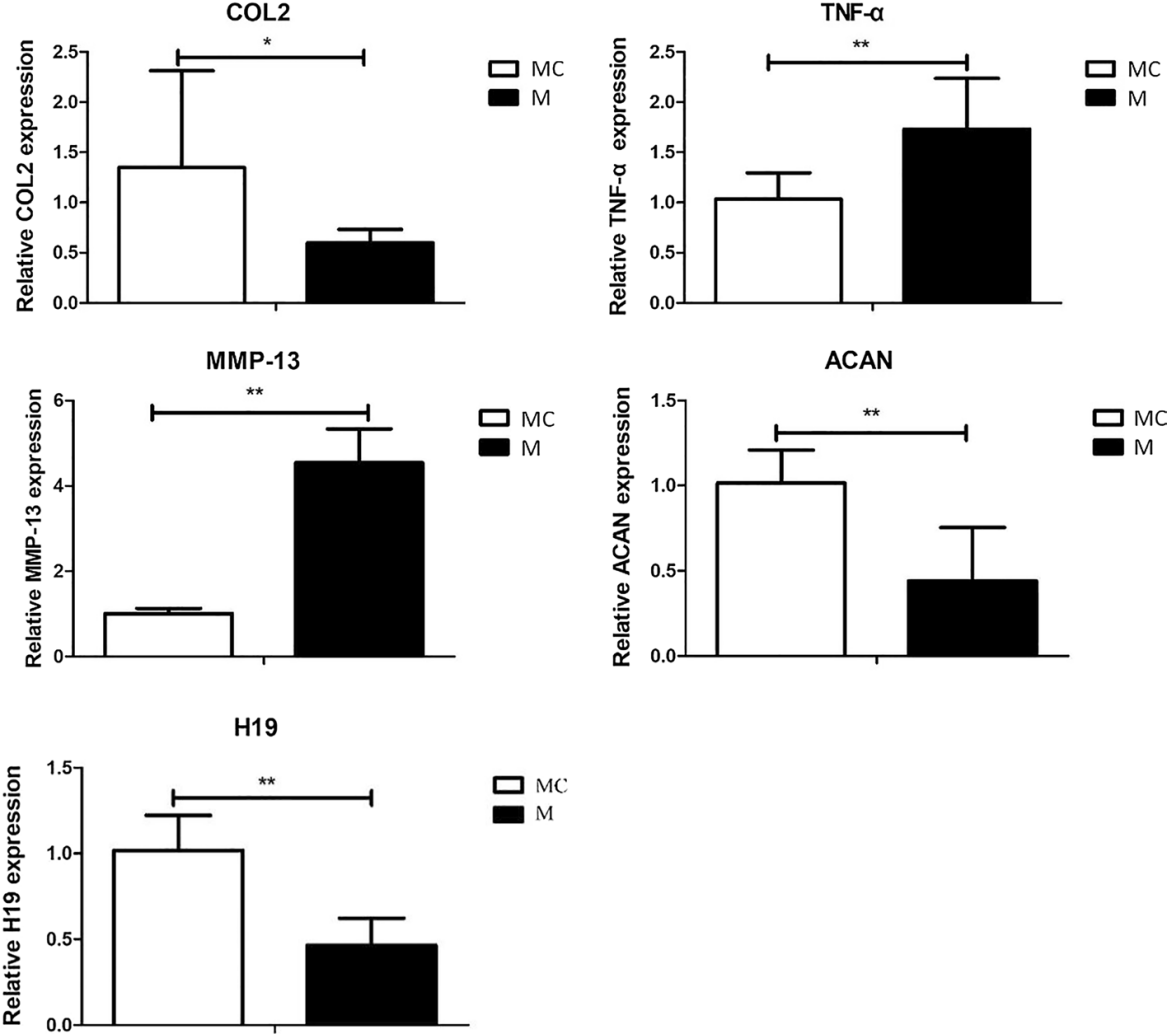
Changes in expression of COL2, ACAN, MMP-13, TNF-α mRNA and lncRNA H19 in mice knee cartilage after high-intensity treadmill running (MC group: model control group M group: treadmill model group, *N* = 6, *P < 0.05, **P < 0.01)

After modeling with high-intensity treadmill running, the total RNA of the right knee cartilage in the MC group and the M group was extracted, and the expression of mRNA levels of the anabolic factors COL2 and ACAN, catabolic factors MMP-13 and proinflammatory cytokines TNF-α and lncRNA H19 in the cartilage were detected using fluorescence quantitative PCR. As shown in Fig. 6, when compared with the MC group, the levels of expression of COL2 and ACAN mRNA in the knee cartilage of the M group decreased significantly, and the levels of expression of MMP-13 and TNF-α mRNA increased significantly after the 4 weeks of high-intensity treadmill running. Meanwhile, the expression of lncRNA H19 in the cartilage of the M group decreased significantly. These results demonstrated that high-intensity treadmill running caused the occurrence of PTOA in mice, and the expression of lncRNA H19 in the cartilage of the PTOA mice was decreased due to the high-intensity treadmill running.

#### Changes in mRNA expression in the knee cartilage in mice after moderate-intensity rehabilitation training

**Fig. 7:**
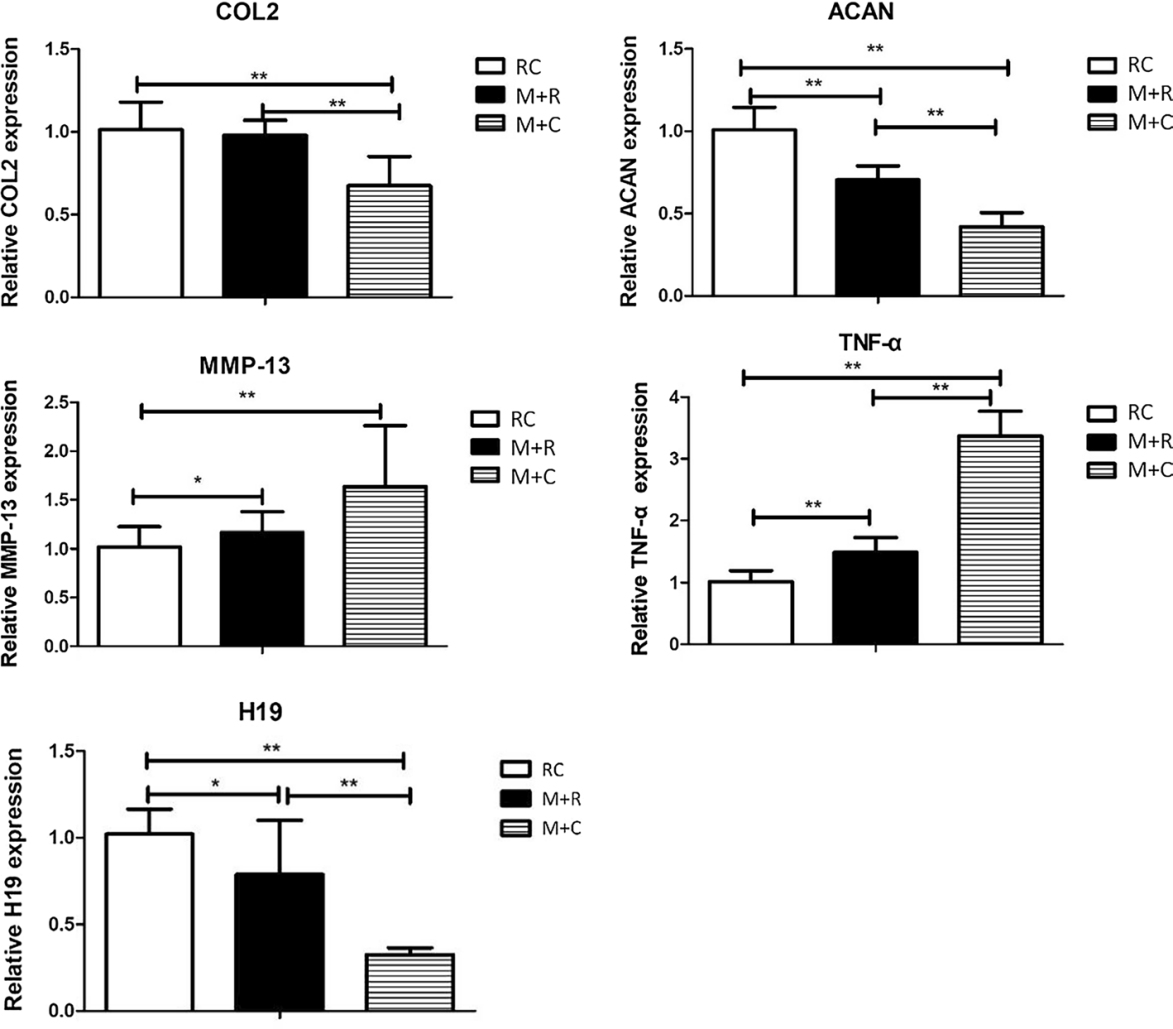
Changes in mRNA and lncRNA H19 expressions of COL2, ACAN, MMP-13, TNF-α, and lncRNA H19 expressions of mice knee cartilage after moderate-intensity treadmill rehabilitation training (RC group: rehabilitation control group, M + R group: treadmill model + rehabilitation training group, M + C group: treadmill model + convalescent group. *N* = 6, *P < 0.05, **P < 0.01)

As shown in Fig. 7, when compared with the M + C group, the levels of expression of COL2 and ACAN mRNA in the knee cartilage of the M + R group increased significantly, and the levels of expression of TNF-α mRNA decreased significantly after 4 weeks of moderate-intensity treadmill rehabilitation training, but there was no significant difference in the decrease of MMP-13 expression. In addition, lncRNA H19 in the M + R group was significantly higher than in the M + C group. When compared with the RC group, the expression of COL2, ACAN, and lncRNA H19 in the cartilage of the M + R group were all reduced, and there was no significant difference in the decrease of COL2, while the expressions of MMP-13 and TNF-α were significantly increased. These results indicated that after the moderate-intensity rehabilitation training, PTOA was alleviated to a certain extent, and the expression of lncRNA H19 was increased.

## Discussion

The mouse model is a commonly used animal model used to study OA [36] and generally includes treadmill running, drug injection, surgery, etc. [37–39]. The mice PTOA model can be successfully induced by treadmill running without the manual operation of the joint cavity, which avoids the difficulty of an operation on the joint cavity and has the advantage of being non-invasive. Therefore, treadmill running is a good modeling method in PTOA manufacturing. Meanwhile, as one of the effective methods to treat OA, exercise therapy has been widely used in clinical practice. Therefore, we used moderate-intensity treadmill exercise as a rehabilitation training method for PTOA mice. Previous studies have shown that exercise therapy can effectively lead to weight loss, thereby reducing joint load and obesity, which is a high-risk factor of OA, and thus alleviate the process of cartilage damage and degeneration [40]. In the present study, we found that the weight of mice showed a continuous downward trend during the 4 weeks of treadmill running, but the average weight loss of mice in the exercise group was within 3 g, indicating that the weight loss is due to the promotion effect of exercise on the health of mice. The slight weight loss has very little effect on the knee joint load, so the influence of weight factors on the progression of PTOA can almost be ruled out in this experiment. In addition, obese mice often exceed the weight of normal mice by more than 20%, thus knee joints will have a significant increase in load, which will aggravate knee cartilage damage. In this experiment, all the mice were fed conventionally, and the weights of mice in the exercise group and the non-exercise group were in the normal range, which provides further confirmation of the weight changes is very little on PTOA progression in this experiment. Therefore, the damage of articular cartilage in MC group mice may only be induced by high-intensity treadmill running and has nothing to do with changes in body weight.

Under normal physiological conditions, the synthesis and degradation of the cartilage matrix maintain a dynamic balance. In OA, this homeostasis is disrupted, the anabolic capacity of chondrocytes is reduced, and the catabolism capacity is increased, which ultimately leads to the degradation of the extracellular matrix [41, 42]. It is generally believed that inflammatory factors such as IL-1β and TNF-α can effectively induce the expression of MMPs and other catabolic enzymes, leading to disorders of extracellular matrix metabolism [43, 44]. Type II collagen is one of the main protein macromolecules that constitutes the extracellular matrix. MMPs are a family of 23 proteases, which have the specific function of hydrolyzing the triple helix structure in type II collagen. Among them, three MMPs (MMP1, MMP3, and MMP13) play a key role in regulating cartilage matrix degradation in OA [45]. In particular, MMP13 plays a central role in the regulation of OA chondrocyte homeostasis and is considered to be the core factor for the irreversible degradation of type II cartilage collagen [46]. In this study, the expression of the inflammatory factor TNF-α in the knee cartilage of high-intensity treadmill running mice was significantly higher than in the blank control group, while the expression of COL2 and ACAN, which represent cartilage anabolism, were significantly down-regulated, and the expression of MMP-13, which represents catabolism, was also significantly up-regulated. Our experiment also conducted rehabilitation training on the PTOA mice through moderate-intensity treadmill running. The results showed that when compared with the PTOA mice in the M + C group, moderate-intensity treadmill running partially reversed the abnormal inflammatory and metabolic indicators.

The histological assessment system is considered to be the gold standard for evaluating OA in mice [47]. Currently, there are a variety of histological scoring methods used to evaluate the degree of OA joint damage [48–50]. In this paper, we used Mankin and OARSI scores to evaluate stained sections of mouse joints. The Mankin scores, first proposed by Mankin et al. [51] in 1971, is the first widely accepted histopathological OA grading system. As a classic OA histological scoring system, it was originally developed to evaluate the histological characteristics of human OA hip joint cartilage, but was subsequently directly or modified and applied to other synovial joints and OA animal models. Mankin scores mainly focus on the structural changes of articular cartilage and tidemarks, as well as the changes in chondrocytes and the content of proteoglycans [52]. However, it should be noted that mouse cartilage is very thin, only a few cell layers thick, and there are no obvious distinguishable superficial, transitional and radial zones, implying that the correlation between the scoring results and OA is suspicious [53]. Therefore, the Mankin score seems to be insufficient for the evaluation of cartilage damage in mice, while the evaluation of rat or human cartilage may be effective [54]. As the Mankin score is limited to the evaluation of cartilage changes and the evaluation efficiency of mouse cartilage damage is not as efficient as that of rats or humans, in our experiments, the OARSI scoring system was used at the same time. The OARSI assessment system focuses on the depth of cartilage defects in the entire joint, including subchondral bone changes in a high-level score. In addition, the OARSI score is a semi-quantitative scoring system, which is relatively easy to apply to experienced and novice raters, with less variability among different raters [47]. Custers et al. [55] concluded that the reliability of the OARSI scoring system is higher than the Mankin score, at least in the evaluation of the OA mouse model. In our experiment, by performing Mankin and OARSI scores on pathological stained sections of mouse knee joints, it was found that the surface of the knee joint in the M group was uneven, with a few cracks and loss of cartilage volume. These results indicate that high-intensity treadmill running successfully induced cartilage lesions in mice. Meanwhile, the Mankin score of the knee cartilage in the moderate-intensity treadmill group was also significantly lower than in the convalescent group, although the reduction of the OARSI score was not statistically significant. The possible reason for the difference in the results of the two evaluation systems is that, as discussed above, the Mankin score focuses on evaluating cartilage damage, while the OARSI score focuses on the depth of cartilage defects involving the entire joint. The motion-induce OA model in our experiment may be mainly manifested in cartilage lesions, so our Mankin score results showed significant differences, while the deep joints of mice such as subchondral bone had no obvious lesions, resulting in the OARSI score results showing only a trend of change and no statistical difference.

LncRNAs are ribonucleic acids that are widely distributed in the human genome and have no protein-coding function. Changes in LncRNAs can cause abnormal expression of genes and lead to various diseases and biological disorders. After using small interfering RNA to knock down the expression of lncRNA H19 in chondrocytes, Zhou et al. [56] found that the proliferation of chondrocytes was significantly inhibited and the apoptosis of chondrocytes increased, while the overexpression of lncRNA H19 could significantly increase the proliferation of chondrocytes. Meanwhile, other studies have found that overexpression of lncRNA H19 can significantly up-regulate the expression of COL2A1 in chondrocytes, while knockout of lncRNA H19 can reduce the expression level of COL2A1 in chondrocytes [57]. These experiments demonstrated from the cellular level that lncRNA H19 may be involved in the regulation of chondrocyte anabolism and apoptosis. Our animal experiment results showed that in the articular cartilage of PTOA mice induced by high-intensity exercise, there was a significantly low expression of lncRNA H19, which indicates that lncRNA H19 may participate in the pathogenesis of motion-induced PTOA. Further studies have shown that four weeks of moderate-intensity treadmill running effectively alleviated the progression of motion-induced PTOA mice. At the same time, the expression of lncRNA H19 in the articular cartilage of moderate-intensity exercise training mice was significantly higher than that of PTOA mice that were raised without exercise intervention. The above results indicate that lncRNA H19 may be closely related to the occurrence and development of motion-induced PTOA mice. It should be noted that the current main modeling method for OA in mice includes surgical induction, collagen induction, and the induction of high-intensity exercise used in this experiment. These different induction methods may involve different pathogenesis. For example, a collagenase injection uses chemical factors to induce chondrocyte apoptosis and matrix degradation, which subsequently leads to subchondral bone disease [58]. Surgical induction induces mechanical homeostasis failure of the joints and results in the loss of mechanical properties of the knee joint and a change in the subchondral bone microstructure, which increases the stress on the overburdened articular cartilage and leads to wear and degradation [59]. Therefore, the regulatory effect of lncRNA H19 on cartilage lesions in the above models may be different than the high-intensity motion-induced PTOA mice model. Further research is needed to fully understand the regulatory effect of lncRNA H19 in the cartilage degeneration of motion-induced PTOA.

As discussed above, OA is an inflammatory joint disease affected by multiple factors, and different pathogenesis results in different times of appearance of cartilage and subchondral bone disease. In OA induced by collagen injection, articular cartilage degeneration first, and then the subchondral bone lesions [58]. In the surgically induced OA model, the microenvironment of the subchondral bone changes first, and then the articular cartilage degrades [59]. In addition, the age-related spontaneous OA mouse models [60] and the ovariectomized mouse OA models [61] can also come to the same conclusion. In our experiments, we tried to evaluate the morphological changes of the subchondral bone to initially explore the possible location of the first lesion in the motion-induced OA model by micro-CT scan. The subchondral bone is in the process of continuous and dynamic bone remodeling, which is regulated by osteoblasts and osteoclasts to ensure the stability of the subchondral bone microstructure. The occurrence of abnormal subchondral bone remodeling is considered to be a typical marker of OA [62]. In the early stage of OA, bone resorption is dominant. The specific process is as follows: abnormal mechanical stress leads to increased bone resorption, and the subchondral bone presents an osteoporotic structure with reduced bone mass, increased trabecular space, and thinning of the subchondral bone plate [63]. In this study, micro-CT scanning of the M group knee joint showed that the BMD, BV/TV ratio, and Tb.N were higher than the MC group. This suggests that high-intensity exercise-induced PTOA mice may be in the early stages of cartilage injury, while there is no obvious morphological change in the subchondral bone. Moreover, after 4 weeks of moderate-intensity treadmill rehabilitation training, the BMD, BV/TV ratio, Tb.Th, Tb.N of M + R group were higher than those in RC group, while Tb.Sp showed the opposite trend, which proved that the subchondral bone of mice did not show the bone resorption phenomenon that may appear in the early stage of OA. Also, in the middle and later stages of OA, bone formation is dominant. The specific process is as follows: to maintain the normal biomechanical properties of subchondral bone, compensatory active bone formation occurs, resulting in the abnormally high conversion of subchondral bone, leading to incomplete mineralization of new bone tissue, even lower than that of healthy people and osteoporosis patients. The manifestation is that the bone mass increases in subchondral bone, and the volumetric BMD decreases, the shape of the trabecular bone is changed from rod shape to plate shape, and the subchondral bone plate is significantly thickened [64]. In this experiment, the micro-CT scan results showed that both BMD and BV/TV of subchondral bone increased after 4 weeks of high-intensity treadmill running, and the trabecular bone did not appear to change shape due to early bone resorption, indicating the subchondral bone of mice did not show the characteristics of late OA at the level of micro-CT. In summary, therefore, we speculate that the subchondral bone of motion-induced OA mice may be in the pre-early stage of OA, neither in the early stage of OA nor in the late stage of OA. A possible cause of this is that mechanical overload on the subchondral bone may be compensated by microcracks. A microcrack is a normal physiological activity of bone cells that is triggered by the mechanical load and is the result of a joint attempting to maintain its integrity, that is, a microcrack may protect the subchondral bone tissue from damage by distributing the mechanical load [65]. The mechanism is that the microcracks of the subchondral bone appear to reduce the elastic modulus of bone tissue, which affects its ability to resist the load of the bone cell. This reduces its ability to absorb mechanical strain and leads to the subchondral bone having a higher mechanical load, resulting in mechanical overload which causes bone matrix damage and activated bone cells to produce the receptor activator of the NF-κB ligand (RANKL). Also, down-regulated osteoprotegerin (OPG) [66] induces osteoclast activation, strengthens bone absorption, and begins bone remodeling to promote bone repair [67]. Therefore, the subchondral bone may compensate for bone injury by early bone remodeling that is associated with microcracks, indicating that motion-induced early OA damage does not first occurs subchondral bone, but in articular cartilage.

Here, we propose a promising research point by reviewing relevant literature and combining our findings. It is well known that lncRNAs act as miRNAs sponges and isolate miRNAs from the targeted coding transcript through shared miRNA binding sites, thereby preventing miRNAs from binding to mRNA targets and ultimately affecting a series of biological processes according to competing endogenous RNA (ceRNA) hypothesis [68]. On the one hand, lncRNA H19 can act as a miRNA precursor to regulate the differentiation of bone marrow mesenchymal stem cells into osteoblasts. On the other hand, lncRNA H19 can act as a competitive endogenous RNA by adsorbing and inhibiting the expression of a variety of miRNAs, thereby participating in regulating osteogenic differentiation (as shown in Table 3). Therefore, we speculate that lncRNA H19 may also participate extensively in the regulation of subchondral bone metabolism during the entire OA process by interacting with miRNAs, due to the uncoupled subchondral bone in OA. Notably, in the middle and late stages of OA dominated by bone formation, the new bone tissue is incompletely mineralized. Matrix mineralization plays a key role in bone formation. Mineralized nodules are formed in osteoblasts at the end of osteogenic differentiation [69]. Research by Wu et al. [70] showed that lncRNA H19 is up-regulated in mineralized osteoblasts and promotes osteoblast matrix mineralization by directly targeting the miR-185-5p/IGF1 axis, indicating that lncRNA H19 may be an effective target for correcting uncoupled bone remodeling in the late stage of OA. In our experiment, we established a specific OA model to preliminarily explore the relationship between lncRNA H19 and the motion-induced OA model. Our results showed that high-intensity mechanical stress reduced the expression of lncRNA H19 in mouse cartilage tissue, and moderate-intensity mechanical stress partially reversed the down-regulation of lncRNA H19 expression. Consistent with our research results, Wang et al. [71] found in an experiment of developmental dysplasia of the hip (DDH) that lncRNA-H19 was down-regulated in DDH patients. The intermittent cyclic mechanical stress (ICMS) cell force model was used to simulate the abnormal biomechanical environment in vitro in this experiment. High-intensity ICMS promoted cartilage degradation and lead to cytoskeletal reorientation. Overexpression of lncRNA H19 inhibited ICMS-induced cartilage degeneration by directly targeting the miR-483-5p/Dusp5 axis. This experiment indicates that lncRNA-H19 may be sensitive to changes in mechanical stress in cartilage. In addition, previous studies established a model of disuse osteoporosis (DOP) with mechanical stress reduction in rats and found that no mechanical load can induce decreased expression of lncRNA H19 in bone tissue and lead to reduced bone formation and DOP through a Wnt signaling cascade [72]. This suggests that lncRNA H19 may be involved in the regulation of mechanical stress-related bone metabolic processes. Unfortunately, our study failed to detect the expression of lncRNA H19 in the subchondral bone of motion-induced PTOA mice, which would have made it possible to determine if there is a difference or correlation between the regulatory effect of lncRNA H19 on bone tissue in DOP and on subchondral bone tissue in motion-induced PTOA. In summary, lncRNA H19 can interact with miRNA to promote osteogenic differentiation of BMSCs and accelerate osteoblast matrix mineralization. It is worth noting that lncRNA H19 may be involved in the regulation of mechanical-related cartilage degeneration and subchondral bone formation. The above research results provide a new perspective on lncRNA and OA for further research. lncRNA H19 may be involved in the regulation of cartilage degeneration and calcification and subchondral bone remodeling in OA by interacting with a variety of miRNAs, and it is reasonable to speculate that lncRNA H19 may be differentially expressed in the early and late stages of OA, at least in the subchondral bone. Further research is needed to confirm the regulation mechanism of lncRNA H19 on OA.

**Table 3:** Interaction between lncRNA H19 and miRNA during osteogenic differentiation

Authors	Regulatory mechanism	Function
He et al. [75]	lncRNA-H19/miR-214-5p/BMP2	Osteogenic differentiation↑
Gan et al. [76]	lncRNA-H19/miR-19b-3p	Osteogenic differentiation↑
Bi et al. [77]	lncRNA-H19/miR-140-5p/SATB2	Osteogenic differentiation↑
Han et al. [78]	lncRNA H19/miR-541-3p/APN	Osteogenic differentiation↑
Li et al. [79]	lncRNA H19/miR-149/SDF-1	Osteogenic differentiation↑
Wu et al. [68]	lncRNA-H19/miR-138/PTK2	Osteogenic differentiation↑
Wang et al. [80]	lncRNA-H19/miR-188/ LCoR	Osteogenic differentiation↑ Adipogenic differentiation↓
Huang et al [81]	lncRNA H19/miR-675/TGF-β1	Osteogenic differentiation↑ Adipogenic differentiation↓
Liang et al. [82]	lncRNA-H19/miR-141/Wnt/β-catenin lncRNA-H19/miR-22/ Wnt/β-catenin	Osteogenic differentiation↑

## Conclusions

High-intensity treadmill running can cause knee cartilage injury in C57BL/6 mice, induce PTOA, and decrease the expression of lncRNA H19 in the articular cartilage. Also, moderate-intensity treadmill rehabilitation training can relieve motion-induced PTOA in mice and increase the expression of lncRNA H19 in the articular cartilage.

## Materials and methods

This study was carried out in strict accordance with the recommendations from the Ethical Committee of Shanghai University of Sport on the Care and Use of Animal Subjects in Research (Approval Number: 2018013), following the protocols approved by the committee. Thirty specific pathogen-free wild-type C57BL/6 male mice (19 weeks old) were purchased from Shanghai Nanfang Biotechnology Co., Ltd. and used in the experiment. The mice were maintained in separate cages in a pathogen-free barrier system animal house at the Shanghai University of Sport. Each of the cages contained five mice. The light inside the barrier was turned on at 8:30 a.m. and turned off at 8:30 p.m., which simulated an alternating day and night for 12 h. The temperature in the animal house was 22 ± 2 ℃, the humidity was 40–70%, and the mice were weighed every Monday.

### Experimental grouping

**Fig. 8:**
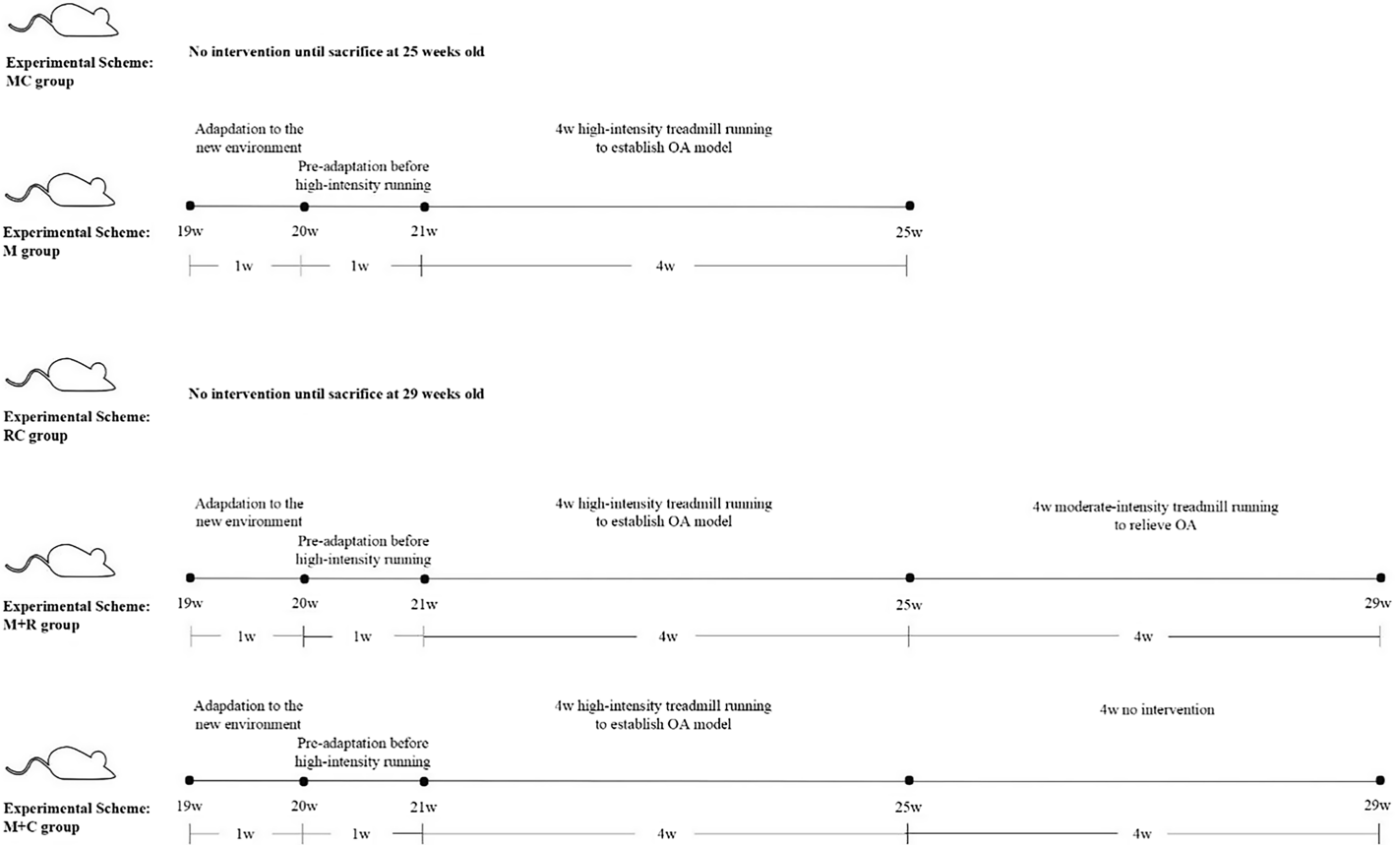
Experimental scheme of mice

All of the mice were acclimated to the new environment for a week. Then they were randomly divided into 5 groups as follows: model control group, (MC group, *n* = 6), treadmill model group (M group, *n* = 6), rehabilitation control group (RC group, *n* = 6), treadmill model + rehabilitation training group (M + R group, *n* = 6) and treadmill model + convalescent group (M + C group, *n* = 6). The mice were 20 weeks old when all of the groups began the experiment. The MC group and the RC group did not receive any intervention, and the remaining three groups (M group, M + R group, and M + C group) were pre-adapted to low-intensity treadmill running for 1 week, and then high-intensity treadmill running for 4 weeks to create a mouse model of PTOA. After 4 weeks of high-intensity treadmill running, samples were collected from the MC group and the M group to test if high-intensity treadmill running can successfully establish a PTOA model. Subsequently, the M + R group participated in 4 weeks of moderate-intensity treadmill rehabilitation training, while the M + C group was conventionally raised in independent cages without any exercise intervention. After 4 weeks of moderate-intensity treadmill running, samples were collected from the RC group, the M + R group, and the M + C group to test the rehabilitation effect of moderate-intensity treadmill running on mice with PTOA, as shown in Table 4 and Fig. 8.

**Table 4:** Grouping scheme of mice

Group	Abbreviation	Intervention scheme	Age at the time of killing
Model control group (*n* = 6)	MC group	No intervention	25w
Treadmill model group (*n* = 6)	M group	4w high intensity	25w
Rehabilitation control group (*n* = 6)	RC group	No intervention	29w
Treadmill model + rehabilitation training (*n* = 6)	M + R group	4w high intensity + 4w moderate-intensity	29w
Treadmill model + convalescent group (*n* = 6)	M + C group	4w high intensity + 4w no intervention	29w

### Treadmill running protocol

According to the literature [73, 74] and our research, the following experimental scheme was developed. The mice began running on the treadmill at 14:00, five times a week, for 80 min each time. The 80 min included 10 min of accelerated motion, 60 min of uniform motion, and 10 min of decelerated motion. During the pre-adaptation period, the uniform motion speed was gradually increased for 5 days. The speed was 10 m/min on the first day, 12 m/min on the second day, 14 m/min on the third day, 16 m/min on the fourth day, and 18 m/min on the fifth day. The gradient of the treadmill was 5°. During the high-intensity treadmill running, the uniform speed was 20 m/min and the treadmill slope was 5°. During the moderate-intensity rehabilitation training, the uniform speed of movement was 8 m/min, and there were no acceleration and deceleration stages included in the scheme before or after the exercise. The exercise duration for this moderate-intensity training was 40 min with no treadmill slope.

### Sampling

When the treadmill running program was completed (at the fifth week for MC and M group; at the ninth week for RC, M + R, and M + C group), the mice were anesthetized by ether inhalation after fasting overnight. After the mice were completely anesthetized, the mice were weighed, and the soft tissues around the knee joint such as the muscles and ligaments were cut off of the lower limbs of the mice with ophthalmic shears, and then the knee joint was cleaned with a PBS solution. The left knee joint was immediately scanned using a micro-CT, and then the samples were placed in the refrigerator with 4% paraformaldehyde solution at 4℃ for 48 h. Next, the samples were placed in the decalcifying solution at room temperature for 30 days. During this time, the decalcifying solution was replaced once a day. The right knee joint was wrapped with gauze, soaked with PBS, and stored in a refrigerator at – 80 ℃ for RNA extraction.

### Paraffin embedding, sectioning, and staining

After the completion of the decalcification process, the knee joint samples were placed in a stainless steel dish and washed with running water for 5 min. The residual soft tissues around the knee joint were carefully trimmed with ophthalmic shears and the unrelated bone was cut around. Only the intact knee joint was retained for paraffin embedding which occurred at a later stage. Then, the knee joints of each group were placed in 50% alcohol at room temperature for 12 h overnight, and the next day the knee joints were dehydrated using gradient ethanol, transparentized with xylene, and embedded in wax. Subsequently, a paraffin embedding machine was used for embedding and the popliteal fossa of the knee joint was aligned to the bottom of the embedding box. The section thickness of the paraffin was 5.0 μm. The paraffin sections were stained according to the standard instructions for hematoxylin–eosin staining and safranin O–fast green staining.

### Histological evaluation

Two widely accepted scoring systems are used to evaluate histological sections to assess the severity of knee OA, including the Mankin histological histochemical grading system and the OARSI histopathology assessment system. The Mankin score is evaluated from four aspects of cartilage structure, cell abnormality, matrix staining, and tidemark integrity, and finally, the sum of the four scores was taken as the comprehensive score. 0 point implies normal cartilage. The higher the overall score, the more severe the OA cartilage lesions. In this experiment, we used the Modified Mankin score due to the more reliable result [52]. In addition, we also used the OARSI grading system to evaluate the pathology of the entire joint in the OA model. The OARSI score focuses on evaluating the depth of cartilage defects. Importantly, the subchondral bone changes are also considered in the higher-level scores. The paraffin sections were scored by two independent observers under a 200-fold light microscope according to the Mankin and OARSI scoring standards. The means of the two groups were used as the final score.

### micro-CT scan

A micro-CT was used to scan the knees of the mice. The scanning parameters were as follows: FOV/diameter: 319,480 μm, voxel size: 10.4 μm, energy: 55 kv, intensity: 72 μm, and sample time: 250,000 μs. When a knee was placed for scanning, we confirmed that the angle of the placement was the same each time, and it was adjusted again in the pre-scan window to ensure the effectiveness and accuracy of the scanning and post-reconstruction. After the scan, 100 layers were selected to reconstruct the medial and lateral tibial plateau of each group from the apex of the tibial plateau, and they were divided into the following groups: MC group lateral tibia plateau (LMC group) and M group lateral tibia plateau (LM), RC group lateral tibia plateau (LRC group), M + R group lateral tibia plateau (LM + R), M + C group lateral tibia plateau (LM + C); MC group medial tibia plateau (MMC group), M group medial tibia plateau (MM group), RC group medial tibia plateau (MRC group), M + R group medial tibia plateau (MM + R group), and M + C group medial tibia plateau (MM + C group). At the same time, micro-CT software was used to analyze the subchondral bone structure. The following measurement parameters were used: (1) trabecular spacing (Tb.Sp); (2) trabecular thickness (Tb.Th); (3) trabecular number (Tb.N); (4) bone volume fraction (BT/TV); and (5) bone mineral density (BMD).

### Real-time PCR detection

The knee joint samples were removed from the – 80 ℃ refrigerator and thawed. The soft tissues around the knee joint, including the joint capsule, were carefully removed and the articular cartilage on the tibial plateau and the femoral condyle were carefully detached. Then, the articular cartilage was put into a small mortar and liquid nitrogen was added to help grind the cartilage into a powder. The cartilage and remaining liquid nitrogen were quickly spooned into the 1.5 ml EP tube before the liquid nitrogen evaporated (if the cartilage was not ground into a powder before the liquid nitrogen evaporated or the powder was not scooped into the EP tube, a small amount of liquid nitrogen should be added again). The total RNA was extracted from the powdered cartilage according to previous studies. Subsequently, SYBR green from the Vazyme Company was used to prepare the corresponding working fluid system according to the given instructions and it was added to a 96-well plate for fluorescence quantitative amplification. With this, the expressions of lncRNA H19, inflammatory factor TNF-α, cartilage anabolic factor COL2, ACAN, and cartilage catabolic factor MMP-13 were detected. β-actin was used as an internal reference gene. The results were analyzed using 2^−△△ct^. The primer premier 5.0 software was used to design all of the primers that were involved in the study. The primer sequences are shown in Table 5.

**Table 5:** Primer sequence

Gene	Primer sequence (5’–3’)
β-actin	Forward: 5’-CTGTCCCTGTATGCCTCTG-3’ Reverse: 5’-ATGTCACGCACGATTTCC-3’
TNF-α	Forward: 5’-AGGCTGCCCCGACTACGT-3’ Reverse:5’-GACTTTCTCCTGGTATGAGATAGCAAA-3’
MMP-13	Forward: 5’ -ACGTGTGGAGGTGAGGCATCC-3’
	Reverse: 5’-GCAGAAGGCAGACCGCAATGG-3’
COL2	Forward: 5’-CAT GAC CTC GTG ATG AAC GTG T-3’
	Reverse: 5’-CGG GTG AGG ACG TTT ACA AAG-3’
lncRNA H19	Forward: 5’-AGGCGAGGATGACAGGTGTGG-3’
	Reverse: 5’-GAACAGACGGCTTCTACGACAAGG-3’
ACAN	Forward: 5’-CACTCCTGCCTGCTATGGAATGC-3’
	Reverse:5’-CCTGGTGATGCTGGTGCTGTTAG-3’

### Data analysis

All of the results were expressed as mean ± standard deviation (X ± SD), and SPSS 20.0 software was used for data analysis and processing. An independent sample T-test was used to compare the MC group and the M group to judge whether or not the PTOA modeling was successful. To judge the recovery effect, the RC group, the M + R group, and the M + C group were compared using a one-way analysis of variance, and a post hoc Bonferroni’s correction for multiple comparisons was used for between-group pairwise comparisons. *P* < 0.05 indicates that the results are statistically different.

## Data Availability

Data sharing does not apply to this article as no datasets were generated or analyzed during the current study.

## Abbreviations

OA: Osteoarthritis
PTOA: Post-traumatic osteoarthritis
lncRNAs: Long non-coding RNAs
lncRNA H19: Long non-coding RNA H19
OPG: Osteoprotegerin
RANKL: Receptor activator of the NF-κB ligand
DOP: Disuse osteoporosis
IL-6: Interleukin-6
TNF-α: Tumor necrosis factor-α
MMPs: Matrix metalloproteinases
Tb.Sp: Trabecular spacing
Tb.Th: Trabecular thickness
Tb.N: Trabecular number
BT/TV: Bone volume fraction
BMD: Bone mineral density
ceRNA: Competing endogenous RNA
